# Cryoglobulinaemic neuropathy: a further cause of bilateral sciatic neuropathy

**DOI:** 10.1186/1755-7682-1-18

**Published:** 2008-10-04

**Authors:** Desireé Pérez, Ricardo Gómez de la Torre, Isabel Carrio, Jesús Pinto, Germán Morís

**Affiliations:** 1 Internal Medicine Service. Hospital San Agustín, Avilés, Asturias, Spain; 2 Pathology Service. Hospital San Agustín, Avilés, Asturias, Spain; 3 Neurology Service. Hospital San Agustín, Avilés, Asturias, Spain

## Abstract

Bilateral sciatic neuropathy is a rare condition and it has been described as a compression or entrapment neuropathy but it is an uncommon clinical manifestation due to necrotizing vasculitis. We report an unusual case of cryoglobulinaemic neuropathy in an elderly woman with no underlying infectious or neoplastic cause; acute bilateral sciatic mononeuropathy was the presenting clinical manifestation of the cryoglobulinaemia.

## Background

The term cryoglobulinaemia refers to the presence in the serum of immunoglobulins (Ig), which precipitate at temperatures below 37°C and redissolve on rewarming. Cryoglobulinemia is not considered a frequent cause of peripheral neuropathy, but it may be more common than previously thought in view of its relationship with the hepatitis C virus (HCV), a widespread infection [1]. The incidence of the cryoglobulinaemic neuropathy (CN) has been described to vary from 6 to 60% [1,2]. The most common form of CN is sensory neuropathy (76%) followed by sensorimotor polyneuropathy (15%) and mononeuritis multiplex (MM) (9%) [3]. We describe an unusual case bilateral sciatic axonopathy in the context of cryoglobulinaemia.

## Case presentation

An 89-year-old woman was assessed for an acute gait disturbance. Her clinical history was unremarkable. The patient had been well until one week before admission; while she was in a sitting position, she developed painless sudden difficulty in moving her feet and abnormal lower extremity sensation. During the following days her neurological picture did not change and no other symptoms appeared. Bowel and bladder function remained normal. She denied radicular pain. She did not recall previous trauma, diarrhoea or febrile illness. On examination, there was a palpable, non-tender, purpuric eruption in the lower limbs and chest which the patient had not noticed. On neurological examination, the patient was alert and oriented. The pupils were equal and reactive, extraocular movements were full. Facial sensation and facial movements were normal. Strength was normal in the upper limbs and decreased in the lower extremities (MRC: extensor digitorum brevis 0/5; extensor hallucis longus 0/5; tibialis anterior 2/5; gastrocnemius 2/5; hamstring muscles 4/5) despite the fact that knee extension and flexion, extension and adduction of the hip were normal. Bilateral sensory loss was detectable below the knee, with sparing of the medial leg. Deep-tendon reflexes were normal but absent in the ankles. Plantar responses were flexor. Romberg's test was negative. The patient was referred for electrophysiologic examination 20 days after the initial symptoms. The results are shown in table 1.

**Table 1:** Patient neurophysiological data.

**RMN**			**LPN**			**RTN**		
		**NV**			**NV**			**NV**

**DL**	4.5 ms	> 4.6 ms	**DL**	4.4 ms	< 4 ms	**DL**	4.8 ms	< 4.5 ms

**MAP**	9 mV	> 8 mV	**MAP**	1.4 mV	> 4.5 mV	**MAP**	3.5 mV	> 5 mV

**MCV**	48 m/s	> 50 m/s	**MCV**	37 m/s	> 43 m/s	**MCV**	41 m/s	> 43 m/s

**SCV**	42 m/s	> 43 m/s						

**SAP**	35 μV	> 36 μV						


**RPN**	**LSN**	**RSN**						
						
ND	ND	ND						
						
			
**EMG**	**RAPB**	**RTA**	**LTA**	**RG**	**LG**			
			
	N	+	+	++	+			

RMN: right median nerve; LPN: left peroneal nerve; RTN: right tibial nerve; RPN: right peroneal nerve; LSN: Left sural nerve; RSN: right sural nerve.

DL: distal latency; MAP: muscular amplitude potential; MCV: motor conduction velocity; SCV: sensory conduction velocity; SAP: sensory amplitude potential.

ND: not detectable. NV: normal values.

RAPB: right abductor pollicis brevis muscle; RTA: right tibialis anterior muscle; LTA: left tibialis anterior muscle; RG: right gastrocnemius muscle; LG: left gastrocnemius muscle.

N: no pathological neurogenic features; +: mild neurogenic features; ++: moderate neurogenic features.

**Figure 1:**
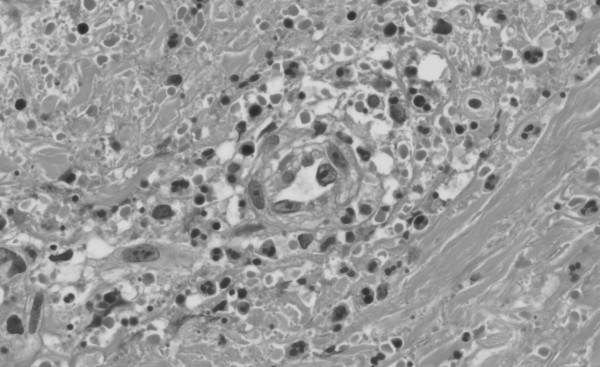
**Pathological examination of the skin lesion**. A leucocytoclastic vasculitis with fibrinoid necrosis of the vessel wall with neutrophilic infiltration and nuclear debris is shown (haematoxylin-eosin, ×100).

Extensive laboratory tests were undertaken. Immmunofixation revealed the presence of cryoglobulin, characterised by polyclonal IgG and monoclonal IgMκ, crycocrit of 3%, circulating rheumatoid factor (RF) (297 kU/L; normal range 0–20 kU/L), hypocomplememtemia C4 (less than 0.08 g/L; normal range 0.15–0.47 g/L) and abnormal liver function test: alanine transaminase 56 UI/L (normal range 4–31 UI/L); alkaline phosphatise 352 UI/l (normal range 35–104 UI/L) and L-lactate dehydrogenase 317 UI/L (normal range 7–32 UI/L). On the other hand, the following test revealed normal or negative results: levels of glucose, glycosylated haemoglobin, renal function, erythrocyte sedimentation rate, haemoglobin, leukocyte count, coagulation tests, C3, Ig, antinuclear antibodies, antineutrophil cytoplasmic antibodies, anti- hepatitis C and B virus antibodies, human immunodeficiency virus antibodies, Lyme antibodies. Cerebrospinal fluid showed normal range for proteins and glucose. Magnetic resonance of the lumbar spine was normal. A computed tomographic scan of the chest, abdomen and pelvis was also unremarkable. The histopathological examination of the skin lesions disclosed findings in keeping with leucocytoclastic vasculitis as is shown in figure 1. Ten days after admission, treatment with oral prednisone with a daily dosage of 1 mg/Kg was initiated. During the following 2 weeks, ankle flexion and extension gradually improved, more so on the left side. The patient was transferred to the rehabilitation service. She died one month later from aspiration pneumonia. An autopsy was not performed.

## Discussion

Unilateral sciatic nerve lesions are common. They are frequently the result of trauma and complications of hip replacement procedures. Conversely, bilateral sciatic neuropathy is a rare condition. It has been described as a compression or entrapment neuropathies, such as heterotopic ossification in a traumatic brain-injured patient [4], after surgery [5,6], due to toilet seat entrapment [7], following sleeping in the lotus position [8], prolonged lying in the supine position with both legs hanging over the end of the bed [9] or being in a prolonged seated position in a floor-level cupboard [10]. Combined distal tibial and peroneal neuropathies have been described following popliteal fossa compression related to legs hanging over a bed footboard [11]. Although there was neither a sural nerve biopsy nor post mortem tissue to confirm, the vasculitic origin is the most plausible cause of the neuropathy. Bilateral sciatic neuropathy is an uncommon clinical manifestation resulting from necrotizing vasculitis. In one series of 94 cases of vasculitic neuropathy, the peroneal nerve or division was involved in 76%, the ulnar nerve in 28%, the tibial nerve in 11% and the median nerve in 9%, but sciatic nerve damage was not reported [12]. In typical cases, the onset of the neuropathy is abrupt and the deficit severe, but in many cases only partial deficit in a nerve territory is observed [13].

The electrophysiological pattern in our case was typically axonal. MM may be the accurate diagnosis and not a length-dependent axonal polyneuropathy. This may be due to the abrupt clinical onset with no subsequent progression and the fact that the median nerve was neither clinically or electrophysiologic affected. It is possible that the initial form of the CN was MM which subsequently evolved into distal symmetrical sensorimotor neuropathy. Although an EMG examination of the biceps femoris was not performed, weakness in hamstring muscles supports the bilateral sciatic nerve damage diagnosis and not that of combined tibial and peroneal neuropathies.

Cryoglobulinaemia is usually classified into three subgroups: type I, single monoclonal Ig, usually a paraprotein, which is almost invariably associated with haematological disorders; types II and III, characterised by polyclonal IgG and monoclonal or polyclonal IgM RF, respectively. Circulating mixed cryoglobulins are often detected in many infectious and systemic disorders. Mixed cryoglobulinaemia (MC) shows a striking association with HCV infection (> 90%) [14]. As a contrast, essential MC is a distinct condition characterised by leucocytoclastic vasculitis and frequent multiple organ involvement with no underlying disorder. The case we describe showed typical manifestation of Type II MC: presence of a monoclonal component and polyclonal Ig, RF activity, low C4, leucocytoclastic vasculitis in purpuric lesions and MM. It has been suggested that monoclonal cryoglobulins are more likely to be associated with demyelinating features, while vasa nervorum vasculitis and the subsequent axonopathy may be more commonly associated with mixed forms [2,15]. On the other hand, the nerve fibre ischemia due to vasa nervorum changes is a consequence of either T-cell-mediated epineural vasculitis or humoral-mediated endoneural microangiopathy [16].

## Conclusion

We present a case of bilateral sciatic neuropathy secondary to cryoglobulinaemia that did not present with an underlying infectious or neoplastic cause.

## Consent

Written informed consent was obtained from the patient for publication of the Case Report and any accompanying images. A copy of the written consent is available for review by the Editor-in-Chief of this journal.

## Competing interests

The authors declare that they have no competing interests.

## Authors' contributions

DP, RGT, IC and GM contributed to the discussion and the writing of the manuscript. JP performed the histological analyses. All authors read and approved the final manuscript.
